# Prosthetic rehabilitation of maxillary lateral incisors agenesis using dental mini-implants: a multicenter 10-year follow-up

**DOI:** 10.1007/s00784-021-04176-0

**Published:** 2021-09-26

**Authors:** M. Lacarbonara, A.P. Cazzolla, V. Lacarbonara, L. Lo Muzio, D. Ciavarella, N.F. Testa, V. Crincoli, D. Di Venere, A. De Franco, D. Tripodi, F.R. Grassi, M. Capogreco

**Affiliations:** 1 Department of Life, Health and Environmental Sciences, Dental Clinic, University of L’Aquila, L’Aquila, Italy; 2 Department of Clinical and Experimental Medicine, University of Foggia, Foggia, Italy; 3 Interdisciplinary Department of Medicine, University of Bari, Bari, Italy; 4 Department of Basic Medical Sciences, Neurosciences and Sensory Organs, University of Bari, Bari, Italy; 5 Department of Medical, Oral and Biotechnological Sciences, University of Chieti-Pescara, Chieti, Italy

**Keywords:** Dental implants, Prosthodontic materials and techniques, Esthetics related to prosthodontics, Implant dentistry

## Abstract

**Objectives:**

Implants are used to replace congenitally missing lateral incisors but often the space across the alveolar crest is too narrow to permit their use. This multicenter study (Dental Clinic of the University of Foggia, Odontostomatology Clinic of the University of L’Aquila) evaluated the efficacy of mini-implants in cases of maxillary lateral incisor agenesis with severe osseous atrophy in 10-year follow-up.

**Materials and methods:**

Forty-seven mini-implants have been inserted in 35 patients affected by lateral incisors agenesis (23 single and 12 bilateral ageneses). All patients underwent orthodontic opening of the space of the upper lateral incisors. After the insertion of the implants, the immediate, non-functional loading, positioning of crowns, presence of pain during percussion and mini-implant function, horizontal and vertical movement when a force of 5 N was applied, ridge loss, and plaque index have been evaluated 1 month after loading, 1 year after loading, and then every 5 years in the following 10 years. Little’s test was used to evaluate the assumption that data of loss to follow-up implants are missing completely at random (MCAR) and that a complete-case scenario could be adopted. Wilcoxon test was carried out to look statistically significant differences between the various parameters resulting in the complete-case scenario and those assumed for the worst scenario. The software R (v. 3.6.1, 2019) was employed to perform the statistical analysis.

**Results:**

The results obtained over 10 years range from 89% of success rate in a worst-case scenario to the 100% using a complete-case analysis with satisfactory values of marginal bone resorption and good conditions of the peri-implant tissue. Ten-year follow-up using complete-case analysis shows survival rates of 100% for implants with no signs of peri-implantitis, stability of the marginal bone levels and soft tissue around the dental implants.

**Conclusions:**

The data collected show very good implant stability, absence of progressive peri-implantitis, and satisfactory aesthetical results in time (no signs of infraocclusion).

**Clinical relevance:**

Mini-implants can be considered a valid and stable over time solution in the restorative treatment of maxillary lateral incisors agenesis.

## Introduction

The prevalence of permanent dentition agenesis is reported to range from 2.2 to 7.6% with a variable prevalence in different ethnic groups and a modest preference toward females [1]. The most commonly reported teeth missing are mandibular second premolars (44%), maxillary lateral incisors (22.9%), maxillary second premolars (21.2%), mandibular central incisors (3.5%) and mandibular lateral incisors (2.5%). Bilateral ageneses of a particular tooth have been highlighted with maxillary lateral incisors being most common (54%) followed by maxillary second premolars (49%), mandibular second premolars (46%) and mandibular central incisors (41%) [2].

According to Bozga et al., the range of agenesis of maxillary lateral incisors is 2.2% to 10.1%[3], whereas Alvesalo and Portin [4] reported a prevalence of maxillary lateral incisor agenesis of 0.52–8.4%, although studies on the Northwestern European population showed a lower congenital prevalence, from 1 to 2% [5].

Dental agenesis is often associated with tooth ectopias and/or other abnormal dental conditions [6, 7] like a smaller or a conoid tooth on the opposite side of the arch. In such cases, the canines are often mesially or lingually positioned, compared with their normal position, the midline being deviated towards the side affected by the agenesis.

Sometimes, agenesis of maxillary lateral incisors is associated with rare diseases or severe syndromes with problems of oral rehabilitation [8–15].

The protocol used for treating agenesis of maxillary lateral incisors depends more on practice organization and the clinical skills available than on considerations regarding treatment effectiveness.

There are at least four options for treating congenital agenesis of the maxillary lateral incisors. These include (1) conservative approach, an aesthetic remodeling of the deciduous maxillary lateral incisor employing a composite resin [16], (2) orthodontic treatment to close the gap with replacement using a reshaped canine [16], (3) orthodontic treatment to open a space with placement of cantilevered resin-bonded fixed dental prostheses [17–19] and (4) orthodontic treatment to open the space with placement of implant-supported fixed prosthetic restorations [17, 20, 21]. The decision for the most adequate treatment setting must consider the type of malocclusion, the anterior teeth relationship, space availability, and the condition of the adjacent tooth [22].

In presence of severe bone atrophy, dental mini-implants may provide a valid solution in patients with narrow alveolar ridges when there is a small interdental space (such as in cases with lateral agenesis).

Mini-implants consist of fixtures whose diameter is between 13/16 and 53/64 inches made up of 5th grade, extra-strong, titanium, sandblasted and acid-etched, with an insertion torque greater than 95 Ncm. At the beginning, they have been used as main fixtures in full-arch implant or prosthetic rehabilitation. In 1999, the Food and Drug Administration (FDA) approved mini dental implants as a safe and permanent option for tooth replacement and they have been used for single-tooth restoration and in cases of a transversal bone deficit.

The hypothesis of this study is to evaluate the efficacy of mini-implants in cases of maxillary lateral incisor agenesis with patients affected by severe osseous atrophy (Type B: breadth 7/64–13/64 in, 25/64–31/64 height, length 7/64–13/64), bone density according to Misch D-3, 350–850 uH B [23] in the long term (10-year follow-up).

## Materials and methods

This multicenter study was conducted from January 2009 to June 2019, in accordance with the provisions of the Declaration of Helsinki at the Dental Clinic of the University of Foggia and the Odontostomatology Clinic of the University of L'Aquila. The study has been approved by the ethics committee of Foggia and L’Aquila (Italy) (protocol number 153-CE-2020/30–11-2020) and informed consent was obtained. Of 46 patients (28 females (61%) age 18.5 ± 1.5 and 18 males (39%) age 19.5 ± 2.2) with lateral incisors agenesis, 16 patients were excluded because 2 finished chemotherapy and 1 radiotherapy recently, 2 presented uncontrolled diabetes mellitus, 1 used corticosteroids and 5 smoke more than 10 cigarettes per day. Five did not carry out periodic controls (2 after the first month, 1 after 1 year and 2 after 5 years) and 30 patients fulfilled the inclusion criteria. Thirty patients (11 men (37%) and 18 (63%) women) affected by maxillary lateral incisor agenesis and submitted to implant replacement were recruited in the study group using complete-case analysis.

**Fig. 1:**
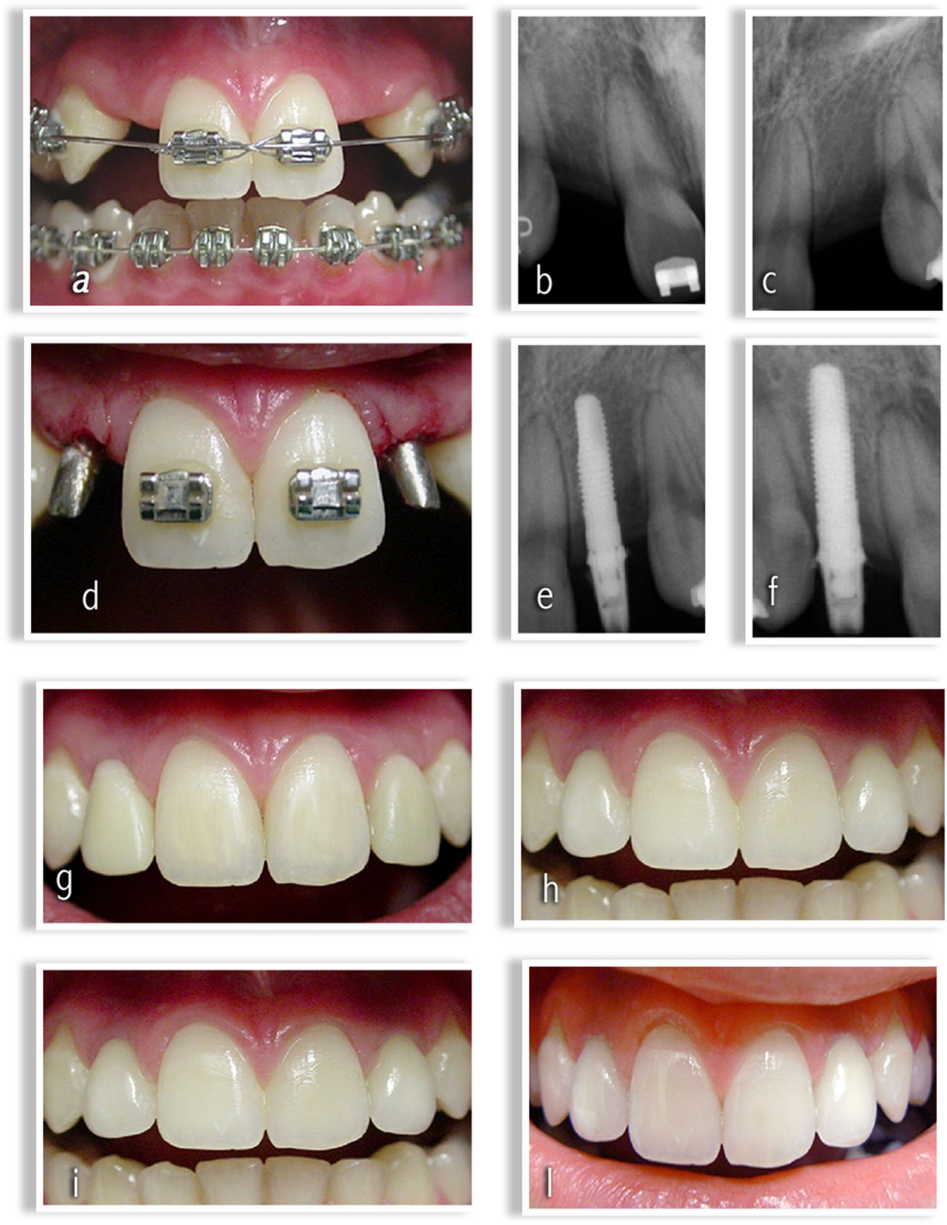
**a** Maxillary lateral incisors agenesis: orthodontic opening of the space in regions 1.2 and 2.2. **b** Radiographic evaluation of alveolar crest in region 1.2. **c** Radiographic evaluation of alveolar crest in region 2.2. **d** Post-surgical implant sites in regions 1.2 and 2.2. **e** Radiographic evaluation post-surgical implant site in region 1.2. **f** Radiographic evaluation post-surgical implant site in region 2.2. **g** Peri-implant tissue aspect after 1 month. **h** Peri-implant tissue aspect after 1 year. **i** Peri-implant tissue aspect after 5 years. **l** Peri-implant tissue aspect after 10 years.

**Fig. 2:**
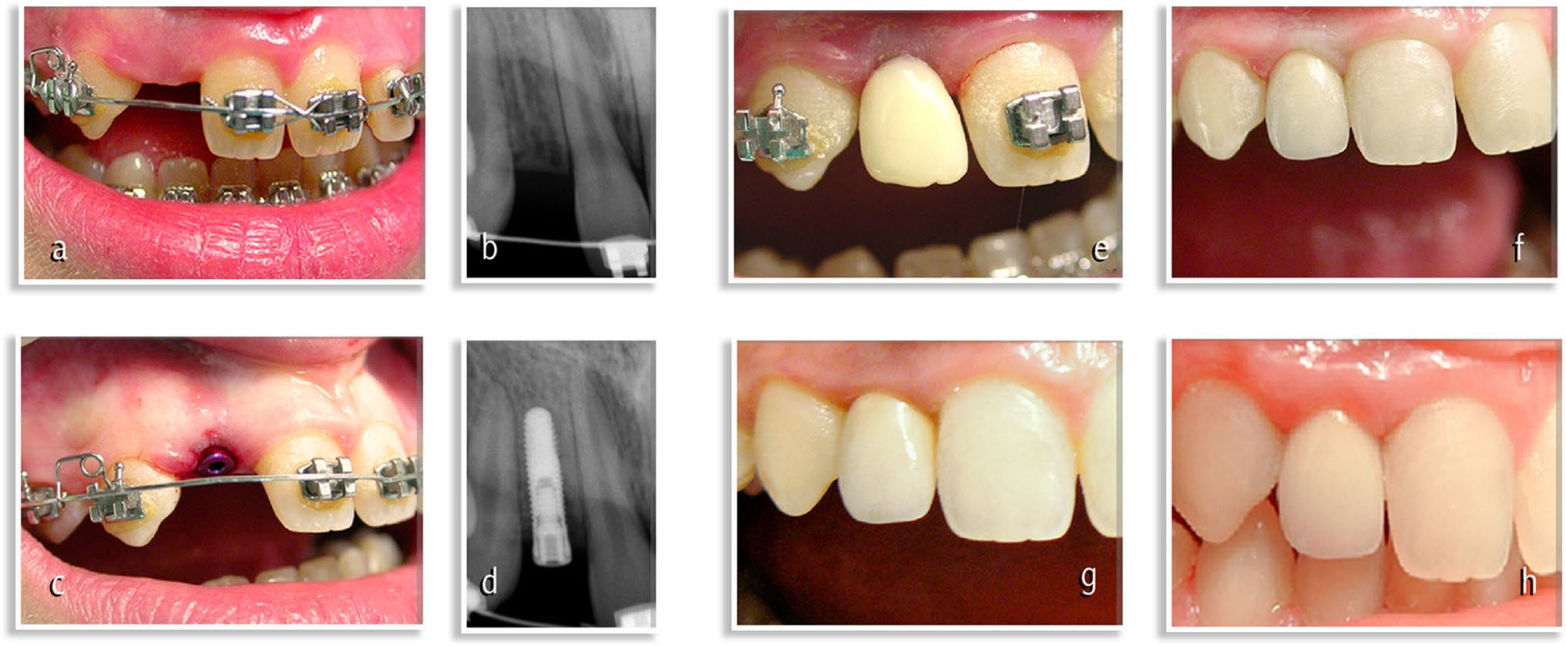
**a** Maxillary lateral incisor agenesis: orthodontic opening of the space in regions 1.2. **b** Radiographic evaluation of alveolar crest in region 1.2. **c** Post-surgical implant sites in regions 1.2 and 2.2. **d** Radiographic evaluation post-surgical implant site in region 1.2. **e** Peri-implant tissue aspect after 1 month. **f** Peri-implant tissue aspect after 1 year. **g** Peri-implant tissue aspect after 5 years. **h** Peri-implant tissue aspect after 10 years.

In 18 patients (11 females and 7 males), the anomaly was unilateral (60%), while in 12 patients (8 females and 4 males), it was bilateral (40%) (Figs. 1, 2).

In this study, 42 mini-implants with a diameter of 2.7 mm and 3 mm (Milo Model, Intra-lock) were examined.

Thirty-five patients fulfilled the inclusion criteria: 14 men (40%) and 21 (60%) women affected by maxillary lateral incisor agenesis and submitted to implant replacement were recruited in the study group.

In 23 patients (13 females and 10 males), the anomaly was unilateral (66%), while in 12 patients (8 females and 4 males), it was bilateral (40%) (Figs. 1, 2).

In this study, 47 mini-implants with a diameter of 2.7 mm and 3 mm (Milo Model, Intra-lock) were examined.

The facial type, the esthetic profile and smile were evaluated clinically [24]. Cephalometric values, molar class, the degree of dentobasal crowding and disharmony, the OVJ and OVB values were evaluated. Further parameters examined were the presence or absence of third molars, position, size and shape of the canines.

The patients included in this study had to respect the following parameters:

- Subscription of informed consent.
- Presence of adjacent dental roots to evaluate the implant site.
- Probable primary implant stability.
- Bone density of type D2 B or D3 according to Mish.
- An average bone thickness from the buccal to the lingual aspect of 4 ± 1 mm.
- The edentulous space, estimated from the distal aspect of the central incisor to the mesial aspect of the canine, measured 5 ± 1 mm.
- Bone height measured about 13 ± 2 mm, estimated from the margin of the alveolar ridge to the floor of the nose.
  Patients with the following criteria were excluded from the study:
- Known or suspected presence of malignant oral cavity pathology.
- Previous clinical history of radiotherapy of the head-neck region.
- History of chemotherapy within 5 years prior to surgery.
- Systemic or local diseases that could have compromised post-operative healing and / or implant osseointegration processes.
- Uncontrolled diabetes mellitus.
- Systematic use of drugs such as corticosteroids or other drugs (antiresorptive or antiangiogenic therapy for the management of osteoporosis and other cancer-related conditions [25–27].
- Smoking 10 or more cigarettes per day.
- Use of alcohol or drugs.
- Absence of obstacles in the implant site (supernumerary teeth, impacted canine, odontomas) [28, 29]
  Forty-two mini-implants with a diameter of 2.7 mm and 3 mm (Milo Model, Intra-lock) were used, as reported below:
- 16 mini-implants 2.7 × 13 mm
- 18 mini-implants 3. × 13 mm
- 8 mini-implants 3 × 11.5 mm
  Forty-seven mini-implants with a diameter of 2.7 mm and 3 mm (Milo Model, Intra-lock) were used, as reported below:
- 18 mini-implants 2.7 × 13 mm
- 20 mini-implants 3. × 13 mm
- 9 mini-implants 3 × 11.5 mm

All the patients underwent orthodontic treatment to open the agenesis space in order to proceed with an implant prosthetic rehabilitation.

The orthodontic treatment has led to achieve the necessary space to put dental implants (Figs. 1a–c and 2a, b).

After the insertion of the implants, the immediate, non-functional loading, and the positioning of porcelain fused to metal (e.g. gold) crowns were performed. Moreover, the following parameters have been evaluated:

- Presence or absence of pain as a consequence of percussion and during the function of mini-implants (Pain) [30];
- Vertical and horizontal mobility in the presence of a force of 5 N (Fix.) [31];
- Amount of alveolar bone resorption through four-point peri-implant probing (i.e. mesial, buccal, distal and palatal—BR) [32];
- Bleeding index according to Loe and Silness (BI) [33, 34];
- Assessment of vertical changes of mini-implants to the adjacent teeth by clinical evaluation (infraocclusion) and intraoral radiographs using method reported in literature [35].

These parameters (Table 1) were followed for a period of 10 years.

**Table 1:** The parameters evaluated

**Parameters**	**Value**	**Description**
A—Pain (ref.28)	0	Absence
	1	Presence at the palpitation, percussion or during the function (failure)
B—Rigid fixation (ref. 29)	0	Absence of visible movements both horizontally and vertically in the presence of a force of 5 N (about 500 g)
	1	From 0 to 0.5 mm of horizontal mobility d.p.c., absence of vertical mobility
	2	More than 0.5 mm of horizontal mobility d.p.c., presence of vertical mobility (failure)
C—Amount of alveolar bone resorption (ref.30)	0	Less than 2 mm of bone crest loss
	1	Loss of bone crest between 2 and 3 mm
	2	Loss of bone crest between 3 and 5 mm
	3	Progressive and uncontrollable bone loss (failure)
D—Bleeding index (Loe and Silness) (ref. 31–32)	0	Absence of bleeding
	1	Bleeding not associated with bone loss (with remission after oral hygiene)
	2	Bleeding associated with bone crest loss (failure)

Follow-up controls were performed 1 month after loading, 1 year after loading and then every 5 years in the following 10 years.

In order to evaluate the efficacy in the long term of mini-implants Wilcoxon test was carried out. In particular, Wilcoxon test was used to evaluate whether there were statistically significant differences between the different parameters taken into consideration in the follow-up. *p*-value threshold of 5% was adopted for the test used. The software R (v. 3.6.1, 2019) was employed to perform the statistical analysis.

Loss to follow-up’s data have been analyzed using Little’s test [36] to evaluate the assumption that data of loss to follow-up implants are missing completely at random (MCAR) [37] and that a complete-case scenario could be adopted.

Patients lost to follow-up been also been the subject of a telephone survey to investigate the reasons for their missing to the follow-up appointments.

In order to evaluate the efficacy in the long term of mini-implants Wilcoxon test was carried out to evaluate statistically significant differences over time (at 1 month, at 1 year, at 5 years and at 10 years) between the parameters mentioned above both in case of a complete-case analysis (in which five implants loss to follow-up have been ignored) that in the worst case scenario (in which implants loss to follow-up are assumed to have all failed).

Finally, Wilcoxon-Mann–Whitney test was carried out to look directly out for any statistically significant differences between the various parameters resulting in the complete-case scenario and those assumed for the worst scenario [38].

*p*-value threshold of 5% was adopted for the tests used. Software R (v. 4.1.0, 2021) was employed to perform the statistical analysis.

### Surgical procedure

On the day of surgery, each patient took antibiotic prophylaxis, with a short-term posology or a single administration of 2 g of Amoxicillin or 600 mg of Clindamycin an hour before the operation. After performing regional loco anesthesia with mepivacaine and adrenaline in a concentration of 1:100,000, a total thickness flap was performed. After the exposure of the bone crest, the implant was inserted using, sequentially, a 1.5 mm diameter pilot drill and then two 2 mm and 2.5 mm diameter drills with sterile external irrigation flow. The implant was taken sterile and placed in the prepared implant site. By means of a surgical contra-angle, the implant was inserted at a speed between 15 and 20 rpm and with a torque not exceeding 35 Ncm. The torque required for final positioning was applied manually by means of a torque wrench (Torque-Lock), thus obtaining a primary stability of 50 Ncm. The abutments were inserted into the surgical site (Figs. 1d and 2c). A single-stitch flap was sutured with a 0000 silk thread. Patients were instructed for home care and rinsed with chlorhexidine-based mouthwash at a 0.20% concentration within 10 days of surgery, twice a day.

### Prosthetic procedure

Immediate non-functional loading was performed on all implants and endoral postoperative control radiography was performed (Figs. 1e–g and 2d, e). The stitches were removed after 10 days from surgery. The final impressions, made of polyether material (Permadyne Penta H, Permadyne Penta L, 3 M ESPE, Seefeld, Germany), were taken 5–7 weeks after operation. The final restorations were realized using Aureo Galvan Crowns (AGC) (Gold bath AGC electroforming, Wieland, Pforzheim, Germany) veneered with feldspathic ceramics (Noritake Super Porcelain EX-3, Noritake Co. Inc., Nagoya, Japan). They were placed 8 to 10 weeks after surgery. Patients were followed for a period of 10 years. Follow-up controls were performed 1 month after loading (Figs. 1g and 2f), 1 year after loading (Figs. 1h and 2f) and then every 5 years (Figs. 1i and 2g) in the following 10 years (Figs. 1l and 2h).

## Results

A total of 30 patients treated with 42 mini-implants were included in this analysis.

A total of 35 patients treated with 47 mini-implants were included in this analysis. The implant has to be surgically inserted into the bone after both the orthodontic therapy and the end of the cranium-facial growth. The patients were treated with one or two implants in positions 1.2, 2.2 and implants were in two different lengths: 13 mm (*n* = 34), 11.5 (*n* = 8), 13 mm (*n* = 38), 11.5 (*n* = 9). Classifications of bone quality were assessed during surgery according to Mish. According to the data of literature, there was a preference toward females (60%). Females (60%) are mostly affected by agenesis, in line with existing medical literature. Osseointegration was achieved for all 42 implants. Osseointegration was achieved for the implants (*n* = 44) which were revised at 1 year. All implants were reported as stable at all follow-up visits after placement of the crown and no implant failure was reported during a 10 year follow-up.

Losses to follow-up, cumulatively calculated per implant, have been 4.2% (*n* = 2) after the first month, 6.4% (*n* = 3) after 1 year and 10.6% (*n* = 5) after 5 years.

Little’s test was applied to missing values for each follow-up appointment showing always a *p*-value greater than 0.05 suggesting that the null hypothesis that the missing data was Missing Completely At Random couldn’t be rejected (Table 2).

**Table 2:** Little’s test showed that the missing data to follow-up implants are missing completely at random (MCAR)

Little test’s results	
Follow-up time	*p*-value
1 month	0.283
1 year	0.272
5 years	0.0829
10 years	0.0829

A telephone survey to investigate the reasons for patients’ missing to the follow-up appointments confirmed the latter hypothesis showing the defection was to be attributable to their relocation (for study in four cases and marriage reasons in the remaining case).

All implants reviewed during follow-up visits (45 at 1 month, 44 at 1 year and 42 at 5 and 10 years) were reported as stable after placement of the crown.

Considering only the 42 implants carried by the patients who completed the 10-year follow-up, no implant failure was reported. No difference was found between male and female patients. No differences related to age were noticed. In the follow-up, no patient experienced any pain; all patients had no visible vertical and horizontal movements; no alveolar bone resorption between 2 and 3 mm occurred. Bleeding was presented in 7 cases (16.6%) after 1 month, in 6 cases (20%) after 1 year, in 5 cases (11.9%) after 5 years and 5 cases (11.9%) after 10 years.

Bleeding was presented in 7 implants after 1 month, in 6 implants after 1 year, in 5 implants after 5 and 10 years.

It was not associated with bone loss and it had a remission after oral hygiene.

Clinical and radiographic evaluations did not show vertical changes of mini implants to the adjacent anterior maxillary teeth in 10-year follow-up because in all the checks carried out, no infraocclusion of the mini-implants was detected. Furthermore, there was no radiological change performed in the distance between the point of reference located on the cervical thread of the implant and the point of reference located on the tooth adjacent to the implant (intersection point between the incisal border and the mesial side of the tooth) projected on the longitudinal axis of the implant in the 10-year follow-up.

Pain and vertical or horizontal mobility in the presence of a force of 5 N did not change over the considered period of 10 years, always reporting a score equal to 0.

Wilcoxon test has been used considering the parameters that changed over time (amount of alveolar bone resorption and bleeding) while no statistical comparison could be performed on pain and vertical or horizontal mobility because their score was null.

Wilcoxon test showed a *p*-value > 0.05 to indicate that there were no statistically significant differences between the parameters taken into consideration(amount of alveolar bone resorption and bleeding) (pain, vertical and horizontal mobility in the presence of a force of 5 N, amount of alveolar bone resorption, bleeding) during the 10-year follow-up nor conducting a case-complete analysis on the remaining 42 implants installed in patients who were regularly followed-up nor considering the worst-case scenario (in which five implants loss to follow-up are assumed to have all failed) (Tables 3 and 4).

**Table 3:** Wilcoxon test, used to compare data adopting the complete-case analysis. Amount of alveolar Bone Resorption (BR) and Bleeding Index (BI) score in 10-year follow-up, showed no statistically significant differences between the parameters taken into consideration (*p*-value > 0.05); *p*-value = 1 when data in two groups are identical

Wilcoxon test (complete-case analysis)	
Follow-up time	*p*-value
Bone resorption (BR)	
1 month vs 1 year	0.32
1 month vs 5 years	0.32
1 month vs 10 years	0.32
1 year vs 5 years	1.00
1 year vs 10 years	1.00
5 years vs 10 years	1.00
Bleeding Index (BI)	
1 month vs 1 year	0.65
1 month vs 5 years	0.41
1 month vs 10 years	0.41
1 year vs 5 years	0.71
1 year vs 10 years	0.71
5 years vs 10 years	1.00

**Table 4:** Wilcoxon test, used to compare data in the worst case scenario Amount of alveolar Bone Resorption (BR) and Bleeding Index (BI) score in 10-year follow-up, showed no statistically significant differences between the parameters taken into consideration (*p*-value > 0.05); *p*-value = 1 when data in two groups are identical

Wilcoxon test (worst case scenario)	
Follow-up time	*p* value
Bone resorption (BR)	
1 month vs 1 year	0.18
1 month vs 5 years	0.06
1 month vs 10 years	0.06
1 year vs 5 years	0.19
1 year vs 10 years	0.19
5 years vs 10 years	1
Bleeding Index (BI)	
1 month vs 1 year	0.74
1 month vs 5 years	0.30
1 month vs 10 years	0.30
1 year vs 5 years	0.42
1 year vs 10 years	0.42
5 years vs 10 years	1

Moreover, Wilcoxon-Mann–Whitney test showed a *p*-value > 0.05, thus indicating that there were no statistically significant differences, comparing the results for the parameters taken into consideration from the complete-case analysis with the data of the worst case scenario (Table 5).

**Table 5:** Wilcoxon-Mann–Whitney test, used to compare the results of the worst case scenario with the ones of the complete-case analysis in a 10-year follow-up, showed no statistically significant differences (*p*-value > 0.05)

Wilcoxon-Mann–Whitney test (worst scenario vs complete case)	
Follow-up time	*p*-value
Bone resorption (BR)	
1 month vs 1 month	0.18
1 month vs 1 year	0.61
1 month vs 5 years	0.61
1 month vs 10 years	0.61
1 year vs 1 year	0.20
1 year vs 5 years	0.20
1 year vs 10 years	0.20
5 years vs 5 years	0.06
5 years vs 10 years	0.06
Bleeding Index (BI)	
1 month vs 1 month	0.70
1 month vs 1 year	0.49
1 month vs 5 years	0.32
1 month vs 10 years	0.32
1 year vs 1 year	0.47
1 year vs 5 years	0.30
1 year vs 10 years	0.30
5 years vs 5 years	0.18
5 years vs 10 years	0.18

## Discussion

The therapeutic plan for congenitally missing lateral incisors requires a complex therapeutic approach aimed at rehabilitating the smile, both in terms of function and aesthetics [39, 40].

Literature describes different treatment options: space closure with mesial repositioning of the canine, followed by tooth recontouring or space opening followed by placement of a prosthesis, transplant or dental implant [41]. Implant placement is considered one of the best solutions in order to obtain an ideal occlusion together with resin-bonded fixed dental prostheses [42, 43]. The orthodontic closure of space, on the other hand, is considered an ideal therapeutic option for adolescent patients as it does not require to wait until the end of the growth period to perform implant surgery as well as for resin-bonded fixed dental prostheses [18, 44].

The closure or opening of spaces in agenesis cases depends on the space discrepancy in the arch, type of facial profile, presence of maxillary dental protrusion or retrusion, type of malocclusion, the presence of dental crowding, agenesis symmetry and size and shape of teeth to be moved. The aesthetic factor is important in the planning of orthodontic treatment for the closure or the opening of the spaces. A bi-protruded or convex profile with protrusion of the upper incisors, a balanced profile with normo-inclined anterior teeth and a minimum or absent space in the arch are indications for the closure of the agenesis space. From the occlusion point of view, class II in neutral occlusion without basal dental discrepancy, class II with basal dental discrepancy treated with inferior extractions, class I with lower crowding, an increased OVJ and an open bite require the closure of the space. The aesthetic analysis of the canine is also necessary: if the canine is mesialized, small, flat with an acceptable color, compatible with the adjacent teeth, the closure of the space is indicated. On the contrary, in the presence of a concave or flat profile, of class III or class I with tendency to class III, of lower diastemas, diminished OVJ, deep bite, global big, well localized and dark-colored canines, it is recommended to open spaces and to plan rehabilitation with implants [45–50].

To obtain correct implant rehabilitation from both aesthetic (a good dental emergence profile) and functional points of view, an adequate bone thickness and an adequate space is necessary. Appropriate space is determined by occlusion, by aesthetics (golden proportion: ideally the lateral incisor should have a width of about two-thirds of the central incisor) and by the distances between the implant and the adjacent teeth that should ideally correspond to about 1.4 mm [49]. Standard-diameter implants require a sufficient width of the alveolar ridge (> 5.5 mm) and the critical condition for good osseointegration is to have an amount of at least 2 mm of healthy bone around the implant [51, 52]. These conditions are not always available in the presence of agenesis of the lateral incisors. Besides, several studies show that the orthodontic opening for the insertion of an endosseous implant generates a reduction in the height and width of the bone crest. Several methods were offered to solve the problem of scant bone thickness in those cases requiring an implant-prosthetic rehabilitation: techniques to change the axis of insertion of the implants, exposing the prosthetic restoration to the concrete risk of failure, techniques of ridge augmentation (guided bone regeneration) with re-absorbable membrane, bone grafting, ridge expansion using split-crest, ERE (edentulous ridge expansion) and RRO (ridge expansion osteotomy). These bone augmentation procedures increase the risk of possible side effects, costs and treatment duration [53].

The data collected in the present study showed that dental mini-implants could be a valid prosthodontic alternative to standard-diameter implants in patients with narrow alveolar ridges and small interdental space such as in cases with lateral agenesis (40–41).

No statistically significant differences (*p*-value > 0.05) were found using Wilcoxon test comparing the parameters taken into consideration (pain, vertical and horizontal mobility in the presence of a force of 5 N, amount of alveolar bone resorption, bleeding) during the 10-year follow-up nor conducting a case-complete analysis on the remaining 42 implants installed in patients who were regularly followed-up nor considering the worst-case scenario (in which five implants loss to follow-up are assumed to have all failed) (Tables 3 and 4).

Even assuming a worst-case scenario, of the 47 implants used, success rates range from 96% (*n* = 45 at the first month) to the 89% (*n* = 42 at 5 and 10 years). Presumed failed implants (five loss to follow-up) were 4% at the first month, 6% at the first year and 11% at 5 and 10 years that is well below the 20% suggested as posing bias by the literature [54].

As the Little test and telephone survey between missing patients suggested that the lost data were Missing Completely At Random, authors are encouraged to consider unbiased the results of the full case analysis [55–57] in a follow-up to 10 years which are equal to 100% (*n* = 42).

Anyway, Wilcoxon-Mann–Whitney test showed a *p*-value > 0.05 thus indicating that there were no statistically significant differences, comparing the results for the parameters taken into consideration from the complete-case analysis with the ones of the worst case scenario (Table 5).

The overall results considered over 10 years range from 89% of success rate in a worst-case scenario to the 100% using a complete-case analysis and are compatible with those found in the literature for shorter periods of time [58, 59].

In this study survival rate of mini-implants in a 10-year follow-up is equal to 100%: no patients experienced pain (*p*-value > 0.05), all mini-implants showed no visible vertical and horizontal movements (*p*-value > 0.05) and no alveolar bone resorption (*p*-value > 0.05). Bleeding was presented in 7 cases after 1 month, in 6 cases after 1 year, in 5 cases after 5 years and 5 cases after 10 years (*p*-value > 0.05).

On the 42 mini-implants kept continuously under observation over 10 years, no patients of the thirty ones who have been followed experienced pain (*p*-value > 0.05), all mini-implants showed no visible vertical and horizontal movements (*p*-value > 0.05) and no alveolar bone resorption (*p*-value > 0.05). Bleeding was presented in 7 implants after 1 month, in 6 cases after 1 year, in 5 cases after 5 years and 5 cases after 10 years (*p*-value > 0.05) (Table 3).

It was not associated with alveolar bone resorption and it had a remission after oral hygiene. Implants showed very good stability over time, absence of inflammation and of clinical or radiographic signs of progressive peri-implantitis. All the abutments were surrounded by healthy and stable peri-implant soft tissues, thanks to the correct design of the final restorations and to the maintenance of good oral hygiene. Aesthetical results on the time are satisfactory with no signs of infraocclusion. In fact, clinical and radiological evaluations did not show vertical changes of mini-implants to the adjacent anterior maxillary teeth in 10-year follow-up in discordance with literature. Several authors reported the presence of infraocclusion for standard size implants probably due to the continuous eruption of adjacent natural teeth and to the growth of the jaws during adolescence and post-adolescence [35, 60–62].

This study, the first to evaluate the behavior of the mini-implants in 10 years, showed no signs of infraocclusion probably thanks to (i) one-piece structure of mini-implants, (ii) to the small diameters of mini-implants that would allow following the movement of the surrounding bone structures and (iii) to the type of osseointegration (mini-implants can be unscrewed with a small torque wrench). Probably, mini-implants contribute to the maintenance of bone properties, bone density, height and width of the alveolar process and they could benefit from the blood supply and uncomplicated healing provided by growing bone [63–65].

Long-term survival studies are lacking and acceptable short-term survival rates (> 90%) of mini-implants are only documented for mandibular overdentures. In a multi-center study, the 4-year survival rate of mini-implants for complete denture stabilization was about 95% without significant differences between the maxilla and mandible [58]. The mean bone loss was insignificantly higher in the maxilla (0.8 mm) than in the mandible (0.5 mm) [66]. In another study on mini-implant supported mandibular overdentures, delayed loading appeared to be preferable to immediate loading regarding implant survival and bone loss [67]. Different studies show that the mean radiographic bone loss rates between 0.4 and 1.2 mm [68].

Medium-term follow-up is documented only in a few case-reports and no studies evaluated marginal bone resorption and the peri-implant tissue conditions with a long-term follow-up [53, 68, 69].

Zarone et al. evaluated the marginal bone resorption and the peri-implant tissue conditions around Narrow-Neck ITI implants in 30 patients by 24–39-month prospective clinical study and present satisfactory values of marginal bone resorption and optimal conditions of peri-implant tissue over time [70]. Rafałowicz et al. assessed the effects of maxillary lateral incisor hypodontia treatment following the use of implantation procedures, fixed and removable dental prostheses, and change in the shape of the canine after a 9 year of follow-up. The results showed that mini-implants with porcelain fused to metal crowns and three-unit porcelain-fused-to-metal fixed partial dentures are the most effective treatment methods [71]. In a review, Gleiznys et al. showed survival rates between 91.17 and 100% in a follow-up duration that ranged from 4 months to 8 years [59].

Mini-implants could be considered a valid therapeutic alternative to resin-bonded fixed dental prostheses and to standard size implants considering follow-up and cost-effectiveness. Data of literature are discordant. While some authors believed that resin-bonded fixed dental prostheses are the optimal solutions in the long term [18, 19], other authors considered resin-bonded fixed dental prosthesis a conservative alternative option wherever possible considering occlusion, state of dentition and tooth conservation as several factors influence its long-term survival (detachment of the restoration, fracture of the porcelain) [72].

Besides, the long-term cost-effectiveness analysis (especially for single-tooth replacement) showed that the cantilevered resin-bonded fixed dental prostheses are not the most cost-effective, long-term option for treatment also considering that the cost of one mini-implants is 3.5 times lower than that of standard size implants [73–75].

## Conclusion

The literature regarding the treatment of maxillary lateral incisor agenesis shows different options: space closure with mesial repositioning of the canine, space opening followed by placement of transplant, prosthesis, resin-bonded fixed dental prostheses or dental implant. This retrospective study, the first to evaluate the behavior of the mini-implants in 10 years, showed a survival rate equal to 100% with satisfactory values of marginal bone resorption and good conditions of the peri-implant tissue.

This retrospective study, the first to evaluate the behavior of the mini-implants in 10 years, showed a survival rate which ranges from 89% in a worst-case scenario to the 100% using a complete-case analysis with satisfactory values of marginal bone resorption and good conditions of the peri-implant tissue.

Mini-implants could be considered a reliable and predictable treatment from an aesthetic, functional and cost-effectiveness point of view. They could be indicated for areas in which the use of implants needs additional bone augmentation/expansion procedures. However, it is necessary to pay particular attention in assessing the bone quality of the implant sites and to maintain good oral hygiene over time to ensure a high success rate.
